# The triglyceride-glucose index as a novel marker associated with sarcopenia in non-diabetic patients on maintenance hemodialysis

**DOI:** 10.1080/0886022X.2022.2128373

**Published:** 2022-10-03

**Authors:** Ruoxin Chen, Liuping Zhang, Mengyan Zhang, Ying Wang, Dan Liu, Zuolin Li, Xiaoliang Zhang, Hui Jin, Bicheng Liu, Hong Liu

**Affiliations:** a Institute of Nephrology, Zhongda Hospital, Southeast University School of Medicine, Nanjing, China; b Institute of Nutrition, Zhongda Hospital, Southeast University School of Medicine, Nanjing, China

**Keywords:** Sarcopenia, triglyceride-glucose index, insulin resistance, non-diabetic, maintenance hemodialysis

## Abstract

**Objective:**

Sarcopenia is a common complication in patients with end-stage kidney disease. Insulin resistance is present in non-diabetic patients undergoing maintenance hemodialysis (MHD) and is an important factor leading to sarcopenia. The triglyceride–glucose (TyG) index, a reliable indicator for evaluating insulin resistance, is widely used in clinical practice. The present study investigated the association between the TyG index and sarcopenia in non-diabetic patients undergoing MHD.

**Methods:**

Relevant clinical data of non-diabetic patients undergoing MHD at our center were collected. The TyG index was calculated using the following formula: ln(fasting triglycerides(mg/dL)×fasting blood glucose(mg/dL)/2). Multivariate logistic regression analyses were used to evaluate the associations. The receiver-operating characteristic curve was used to analyze the predictive value of the TyG index in sarcopenia.

**Results:**

Of the 142 patients undergoing MHD who were included, 75 (52.82%) were men, the mean age was 54.05 ± 13.97 years, and 40 (28.17%) patients satisfied the diagnostic criteria for sarcopenia. The TyG index of participants with sarcopenia was higher compared with those without sarcopenia (8.83 ± 0.45 vs. 8.49 ± 0.50, *p* < 0.001). The prevalence of sarcopenia increased with increasing TyG index tertile (T1, 8.51%; T2, 31.91%; T3, 43.75%; *p* = 0.001). Logistic regression analysis indicated that the TyG index was an independent risk factor for sarcopenia (odds ratio, 4.21 [95% confidence interval, 1.85–9.59], *p* = 0.001).

**Conclusion:**

A higher TyG index was associated with an increased risk of sarcopenia in non-diabetic patients undergoing MHD; it may be used as a novel marker to reflect the presence of sarcopenia.

## Introduction

In recent years, the incidence of end-stage kidney disease (ESKD) has been increasing annually, with poor prognosis and high cost [1]. Maintenance hemodialysis (MHD) is the main alternative therapy for ESKD, and approximately 86% of patients with ESKD in China choose it [2]. According to the China Kidney Disease Network, the prevalence of hemodialysis in China was approximately 402.18 per million population in 2015, increasing by more than 11% per year [3,4]. Approximately 3.9–63.3% of patients undergoing MHD have sarcopenia [5,6]. As a common complication in patients undergoing MHD, sarcopenia seriously endangers human health and aggravates the economic burden worldwide [7,8].

Sarcopenia, a progressive and generalized skeletal muscle disorder, is associated with an increased risk of negative outcomes, including physical disability, falls, fractures, and mortality [8]. Skeletal muscle is the predominant site of insulin-mediated glucose uptake in the postprandial state [9], and insulin resistance is an important pathophysiological factor leading to its loss [10]. Insulin resistance is prevalent in nondiabetic patients with ESKD, and the influencing factors, such as uremic toxin accumulation, metabolic acidosis, and microinflammatory state, are complex [11–13]. The triglyceride-glucose (TyG) index, calculated from fasting glucose and triglyceride levels, has been proposed as a reliable and convenient indicator for evaluating insulin resistance [14,15].

Given the close relationship between insulin resistance and sarcopenia, the elevation of the TyG index may help identify sarcopenia. However, few studies have explored the association between the TyG index and sarcopenia in non-diabetic patients undergoing MHD. Therefore, we investigated this relationship, hoping that effective interventions can be implemented in such patients in a timely manner.

## Methods

### Study design and participants

This was a single-center, cross-sectional study. Patients who underwent regular hemodialysis at our hospital were selected as study subjects. Inclusion criteria were as follows: (a) age >18 years, (b) stable hemodialysis for more than 3 months, and (c) regular hemodialysis three times a week, 4 h each session. The exclusion criteria were as follows: (a) diagnosis of diabetes mellitus by doctors; (b) presence of malignant tumors, severe heart failure (NYHA IV), acute severe infection in the past 3 months, connective tissue disease, severe metabolic diseases, decompensated chronic liver disease, hematological diseases, etc.; (c) inability to complete the handgrip strength test or the 6-m walk test; (d) presence of severe cognitive impairment or mental illness; (e) pregnancy or lactation; and (f) lack of complete data. Informed consent was obtained from all the patients.

This study was approved by the Independent Ethics Committee (IEC) for Clinical Research of Zhongda Hospital, affiliated with Southeast University (batch number:2021ZDSYLL230-P01). This research was conducted in accordance with the principles of the Declaration of Helsinki.

### Clinical data

Face-to-face interviews were conducted to verify demographics (age and sex) and clinical characteristics (presence of hypertension, tobacco use, and duration of dialysis). Blood samples were collected in the morning after overnight fasting. Clinical data (fractional clearance index for urea [Kt/V], blood glucose, triglycerides, cholesterol, high-density lipoprotein cholesterol [HDL-C], low-density lipoprotein cholesterol [LDL-C], hemoglobin, albumin, calcium, phosphorus, C-reactive protein [CRP], and parathyroid hormone [PTH]) of the preceding 3 months were collected from electronic medical records. Demographic data were collected by dietitians and graduate students. Two clinical medical staff checked patients’ electronic medical records for relevant data. The TyG index was expressed as ln(fasting triglycerides(mg/dL)×fasting blood glucose (mg/dL)/2) [15]. Professionals in the nutrition department collected the malnutrition-inflammation score (MIS) results of the participants.

### Indicators of sarcopenia

Body composition was measured using multi-frequency bioelectrical impedance analysis (BIA) (InBody 770; InBody Co., Ltd. Korea) within 30 min of dialysis completion. Measurements included the skeletal muscle index (SMI), arm muscle circumference (AMC), and protein. The SMI was defined as appendicular skeletal muscle mass divided by height squared. The muscle strength of the subjects was assessed by measuring handgrip strength (kg) on the non-fistula side. Subjects were seated in an upright position, with their feet naturally on the ground, hip flexion at 90°, elbow flexion at 90°, upper arm flat with chest, forearm positioned neutrally, and wrist extension at 0°–30°. The maximum value was obtained by measuring the non-fistula side three times. The physical performance of the subjects was assessed by a 6-m walking test, and the step speed was measured using a stopwatch three times. The fastest step speed was taken for statistical purposes [16].

### Diagnosis of sarcopenia

According to the 2019 revised Asian Working Group for Sarcopenia (AWGS) criteria, cutoff values for these diagnostic components of sarcopenia were as follows: (1) low muscle strength was defined as dominant handgrip strength <28 kg for men and <18 kg for women; (2) low muscle mass, measured using BIA, was defined as SMI <7.0 kg/m^2^ for men and <5.7 kg/m^2^ for women; (3) the 6-meter walking test <1.0 m/s was recommended for the evaluation of physical ability; (4) the diagnosis of sarcopenia was based on the 2019 AWGS criteria, with low muscle mass accompanied by either low muscle strength or low physical performance [16].

### Malnutrition-Inflammation score

MIS is mainly used to evaluate the nutritional status of patients with chronic kidney disease (CKD), especially ESKD. It contains ten elements from four sections: (1) past medical history, including weight change in edema-free post-hemodialysis body weight in the past 6 months, dietary intake, gastrointestinal symptoms, functional capacity, and comorbidities, including duration of dialysis; (2) physical examination of subcutaneous body fat and signs of muscle wasting according to the subjective global assessment (SGA) criteria; (3) body mass index (BMI); and (4) laboratory parameters, including serum albumin level and total iron binding capacity. The sum of all 10 components results in an overall score ranging from 0 (normal) to 30 (severely malnourished) [17,18]. Assessments were performed by well-trained dietitians.

### Statistical analyses

Normally distributed continuous variables are expressed as mean ± standard deviation and compared using Student’s *t*-test or one-way analysis of variance (ANOVA). Non-normally distributed continuous variables are expressed as median and quartile spacing, and the Mann–Whitney *U* test was used for comparison between groups. Categorical variables are expressed as counts and percentages and were compared using the chi-square test. Univariate and adjusted multivariate logistic regression analyses were used to analyze the relationship between the TyG index and sarcopenia. Receiver-operating characteristic (ROC) curve analysis was performed to evaluate the diagnostic value of the TyG index for sarcopenia. The best cutoff value was calculated using the Youden index. Statistical analysis was performed using SPSS software (version 26.0, SPSS, Chicago, IL, USA). A value of *p* = 0.05 was considered statistically significant. Furthermore, for figures, Prism version 8.0 (GraphPad Software, La Jolla California, USA) was used.

## Results

### General information

A total of 142 non-diabetic patients undergoing MHD at our center were included in this study. Forty (28.17%) patients met the diagnostic criteria for sarcopenia. Compared with the non-sarcopenia group, the sarcopenia group was older (62.10 ± 13.47 years vs. 50.89 ± 12.90 years, *p* < 0.001) and had a lower BMI (20.75 [18.43–22.00] kg/m^2^ vs. 22.40 [20.38–25.05] kg/m^2^, *p* < 0.001). There were no statistical differences between the two groups in terms of blood pressure, duration of dialysis, Kt/V, CRP, PTH, or other parameters (Table 1).

**Table 1.** Characteristics of study participants.

	All patients	Sarcopenia	Non-Sarcopenia	*p*-Value
Number of participants, *n* (%)	142	40 (28.17%)	102 (71.83%)	
Sex (male/female), (*n*)	75/67	23/17	52/50	0.484
Age, (year)	54.05 ± 13.97	62.10 ± 13.47	50.89 ± 12.90	<0.001
BMI, (kg/m^2^)	21.85 (19.50–23.93)	20.75 (18.43–22.00)	22.40 (20.38–25.05)	<0.001
Tobacco use, *n* (%)	34 (23.94%)	9 (6.34%)	25 (17.61%)	0.801
Hypertension, *n* (%)	131 (92.25%)	37 (26.06%)	94 (66.20%)	0.945
SBP, (mmHg)	142.03 ± 21.28	143.10 ± 23.75	141.61 ± 20.33	0.708
DBP, (mmHg)	82.99 ± 13.13	82.37 ± 14.44	83.23 ± 12.64	0.730
Fasting plasma glucose, (mmol/L)	4.76(4.39–5.13)	5.07 ± 0.80	4.71 ± 0.60	0.004
Triglyceride, (mmol/L)	1.44 (0.97–1.96)	1.66 (1.24–2.20)	1.27 (0.91–1.82)	0.001
TyG index	8.58 ± 0.51	8.83 ± 0.45	8.49 ± 0.50	<0.001
Total cholesterol, (mmol/L)	4.13 (3.41 ± 4.83)	3.95 (3.09–4.48)	4.16 (3.51–4.89)	0.239
HDL-C, (mmol/L)	1.15 ± 0.28	1.13 ± 0.24	1.16 ± 0.29	0.624
LDL-C, (mmol/L)	2.38 ± 0.76	2.29 ± 0.64	2.42 ± 0.81	0.344
Hemoglobin, (g/L)	106.65 ± 17.31	106.80 ± 19.16	106.59 ± 16.63	0.948
Albumin, (g/L)	40.75 (37.68–42.80)	39.20 ± 4.72	40.70 ± 3.75	0.049
Calcium, (mmol/L)	2.28 ± 0.28	2.31 ± 0.28	2.26 ± 0.27	0.327
Phosphorus, (mmol/L)	1.87 ± 0.54	1.71 ± 0.45	1.93 ± 0.55	0.022
CRP, (mg/L)	2.07 (0.82–6.58)	2.30 (0.82–6.60)	1.96 (0.82–6.15)	0.552
PTH, (pg/mL)	331.72 (167.76–587.30)	247.25 (118.11–542.76)	347.69 (184.59–595.52)	0.314
Duration of dialysis, (month)	88.92 (49.04–137.84)	80.38 (46.23–112.03)	91.03 (52.64–144.02)	0.334
*Kt*/V	1.25 (1.23–1.27)	1.25 (1.23–1.27)	1.25 (1.22–1.27)	0.120
HS, (kg)				
Men	30.44 ± 12.20	22.57 ± 6.62	33.93 ± 12.52	<0.001
Women	19.91 ± 8.72	13.84 ± 5.62	21.98 ± 8.65	0.001
6-metre walking speed, (m/s)	0.95 ± 0.23	0.88 ± 0.29	0.97 ± 0.20	0.032
SMI, (kg/m^2^)				
Men	7.29 ± 0.97	6.41 ± 0.44	7.67 ± 0.89	<0.001
Women	5.97 ± 0.73	5.12 ± 0.36	6.26 ± 0.58	<0.001
AMC, (cm)				
Men	27.15 ± 2.82	24.94 ± 1.43	28.13 ± 2.73	<0.001
Women	24.38 ± 2.21	22.75 ± 1.55	24.93 ± 2.14	<0.001
Protein, (kg)	8.70 (7.50–10.00)	7.80 (6.65–8.95)	9.15 (7.70–10.63)	<0.001
MIS	13.00 (11.00–14.00)	13.00 (12.00–16.00)	12 (10.75–14.00)	0.004

BMI: body mass index; SBP: systolic blood pressure; DBP: diastolic blood pressure; TyG index: triglyceride glucose index; HDL-C: high-density lipoprotein cholesterol; LDL-C: low-density lipoprotein cholesterol; CRP: C-reactive protein; PTH: parathyroid hormone; *Kt*/V: fractional clearance index for urea; HS: handgrip strength; SMI: skeletal muscle index; AMC: arm muscle circumference; MIS: malnutrition-inflammation score.

Participants with sarcopenia showed higher TyG indices (8.83 ± 0.45 vs. 8.49 ± 0.50, *p* < 0.001). In addition, we found that compared with the non-sarcopenia group, the sarcopenia group scored significantly higher in MIS (13.00 [12.00–16.00] vs. 12.0 [10.75–14.00], *p* = 0.004), had significantly lower protein (7.80 [6.65–8.95] vs. 9.15 [7.70–10.63], *p* < 0.001), and had significantly lower albumin (39.20 ± 4.72 vs. 40.70 ± 3.75, *p* = 0.049) (Table 1).

### Association between TyG index and sarcopenia

**Figure 1.**
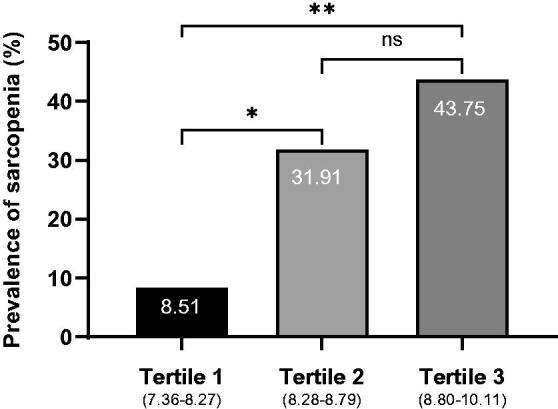
Prevalence of sarcopenia according to TyG index tertiles in non-diabetic patients on maintenance hemodialysis. Significant differences between groups are indicated by *(*p <* 0.05) and **(*p <* 0.001), and ns indicates no significant difference (*p >* 0.05).

To further confirm our conclusions, we divided the TyG index into three groups according to tertiles. As shown in Figure 1, there were significant differences between the three groups. The prevalence of sarcopenia increased with increasing TyG index tertile (Figure 1).

In the univariate analysis, the TyG index was significantly associated with sarcopenia (odds ratio [OR], 4.21; 95% confidence interval [CI], 1.85–9.59; *p* = 0.001). In multivariate analysis, the results were statistically significant in the adjusted models. As shown in Table 2, after adjusting for age, BMI, hemoglobin, albumin, calcium, phosphorus, CRP, AMC, protein, and MIS, multivariate analysis showed that the TyG index was positively associated with sarcopenia (OR 9.97; 95% CI, 2.90–34.23; *p* < 0.001).

**Table 2.** Multivariate logistic regression analysis of sarcopenia with the TyG index.

TyG index	*B*	*SE*	*OR*	95% CI	Wald	*p*-Value
Unadjusted	1.44	0.42	4.21	1.85–9.59	11.67	0.001
Model 1	1.61	0.45	4.98	2.05–12.09	12.61	<0.001
Model 2	2.02	0.51	7.51	2.74–20.58	15.38	<0.001
Model 3	2.30	0.63	9.97	2.90–34.23	13.33	<0.001

BMI: body mass index; CRP: C-reactive protein; AMC: arm muscle circumference; MIS: malnutrition-inflammation score.

Model 1: Age, Sex.

Model 2: Age, Sex, BMI, Tobacco use, Hypertension.

Model 3: Age, BMI, Hemoglobin, Albumin, Calcium, Phosphorus, CRP, AMC, Protein, MIS.

**Figure 2.**
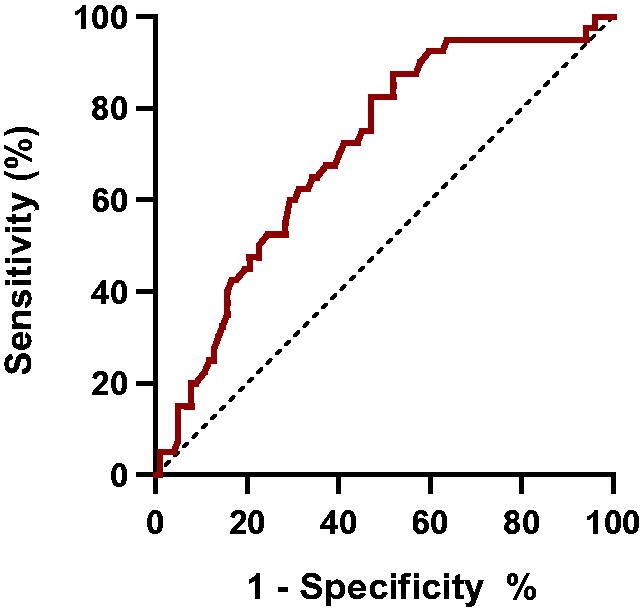
Receiver-operating characteristics (ROC) curves of the TyG index (AUC = 0.707).

Since the TyG index was an independent marker of sarcopenia, we used ROC curves to explore whether combinations of variables could act as diagnostic markers to identify sarcopenia in non-diabetic patients undergoing MHD. According to the ROC curve analysis, the area under the curve for the TyG index was 0.707 (*p* < 0.001) (Figure 2). The cutoff value of the TyG index was 8.39 with a sensitivity of 87.50% and specificity of 48.00%.

## Discussion

This cross-sectional survey among non-diabetic patients undergoing MHD investigated the association between the TyG index and sarcopenia. After adjusting for confounders, multivariate analysis results demonstrated that the TyG index was independently and positively correlated with sarcopenia. ROC analysis revealed that the TyG index was a useful marker for predicting sarcopenia in non-diabetic patients undergoing hemodialysis. Moreover, patients undergoing MHD with sarcopenia had higher MIS scores and poorer nutritional status than those without sarcopenia.

Kidneys are recipient organs of signaling molecules from skeletal muscle mass [19]. There are two main mechanisms for muscle mass loss in CKD: (1) increased muscle catabolism *via* the activation of, among others, the ubiquitin-proteasome system, caspase-3, and lysosomes pathways; (2) impaired muscle growth, regeneration, repair, and suppression of protein synthesis with components of abnormal myogenesis [20]. The development of sarcopenia in patients undergoing MHD is mainly due to the alteration of multiple factors that regulate the balance between catabolism and anabolism in muscle, probably due to the uremic environment caused by ESKD [21]. The factors of sarcopenia in ESKD are complex, including insulin resistance, inflammation, malnutrition, abnormal myogenic regulatory factors, increased myostatin, and decreased physical activity [22–24]. Under the influence of multiple factors, the persistent imbalance between muscle anabolism and catabolism can lead to protein degradation and muscle atrophy, which in turn affects muscle structure and function [25,26]. Notably, the mechanisms of sarcopenia in dialysis patients are complex and remain a research direction worthy of future consideration.

Insulin resistance is an independent risk factor for CKD [27] and is associated with skeletal muscle protein breakdown [22,28]. Thomas et al. [29] found that CKD-induced inflammatory cytokines stimulate nuclear factor (NF)-κB activation, allowing increased expression of signal regulatory protein-α (SIRP-α) proteins. In addition, SIRP-α interacts with insulin receptors and insulin receptor substrate-1, which can reduce tyrosine phosphorylation, ultimately impairing intracellular insulin signaling and inducing muscle wasting. Moreover, the accumulation of uremic toxins, metabolic acidosis, and persistent micro-inflammatory state are unique factors that aggravate insulin resistance in patients with CKD [11,13]. Given the close pathophysiological relationship between sarcopenia and insulin resistance [10,28,30], the association between the TyG index and sarcopenia in patients undergoing MHD is plausible.

The TyG index is a reliable indicator for evaluating insulin resistance and is associated with sarcopenia in ESKD patients [31,32]. In 2020, Ahn et al. [33] conducted a cross-sectional study demonstrating that the TyG index was associated with low muscle mass in non-diabetic adults without CKD. Our study provides new findings regarding the relationship between the TyG index and sarcopenia in non-diabetic patients undergoing MHD. The results showed that the TyG index was an effective index to reflect the presence of sarcopenia and had a guiding value. Our logistic regression analysis suggested that in non-diabetic patients undergoing MHD, the TyG index was independently associated with sarcopenia factors and may reflect sarcopenia. For each unit increase in the TyG index, the risk of sarcopenia increased 4.21-fold. In addition, the area under the ROC curve was 0.707 (*p* < 0.001), indicating that the TyG index had some diagnostic value for sarcopenia in non-diabetic patients undergoing MHD.

Malnutrition is prevalent among patients undergoing MHD. Nutrients, such as protein and fat, are constantly consumed as CKD progresses, resulting in malnutrition in patients [34,35]. Approximately 23–94% of MHD patients are malnourished due to the synergism of nutritional intake, loss of appetite, inflammation, electrolyte balance disorders, and other factors [36]. In our study, we found that patients with sarcopenia had significantly lower protein levels and AMC than those without sarcopenia. In addition, plasma albumin levels are significantly reduced in patients with sarcopenia. To further understand the nutritional status of patients, we conducted an investigation using the MIS. MIS incorporates objective laboratory parameters based on the SGA and is an effective screening tool for assessing malnutrition and quality of life in patients undergoing MHD [17,37–39]. Our results showed that among non-diabetic patients undergoing MHD, those with sarcopenia had higher MIS scores, suggesting that their nutritional status was relatively poor. Insufficient nutrient intake can cause multi-system dysfunction in the body, which increases the risk of muscle reduction, falls, and mortality [40]. Adopting appropriate nutritional interventions can improve the adverse state of patients, promote the synthesis of muscle proteins, reduce the occurrence of sarcopenia, and delay its progression [41–44]. The MIS can reflect the nutritional status of non-diabetic patients undergoing MHD, which has clinical value in the treatment of sarcopenia.

Despite the efforts made in this study, there are still several limitations that should be mentioned. First, due to the limitation of conditions, the sample size of this study was small, and the research subjects were all from one center. Therefore, multi-center studies with larger sample sizes should be considered for future studies. Second, this was a clinical cross-sectional study, and we were unable to conclude a cause-and-effect relationship.

## Conclusions

To the best of our knowledge, our study is the first to investigate the relationship between the TyG index and sarcopenia in non-diabetic patients undergoing MHD. The results of this study showed that a higher TyG index was associated with an increased risk of sarcopenia in non-diabetic patients on MHD and may be used as a novel marker to reflect the presence of sarcopenia. Furthermore, patients with sarcopenia tended to have higher MIS scores; thus, timely nutritional intervention measures should be implemented to improve prognosis. These findings should be interpreted with caution, and further longitudinal studies are needed to elucidate the causal relationship between the TyG index, MIS, and sarcopenia.

## Data Availability

The data analyzed in this study are available from the corresponding author (Hong Liu, e-mail: jstzliu@sina.com) upon reasonable request.
